# The role of Src family kinases in growth and migration of glioma stem cells

**DOI:** 10.3892/ijo.2014.2432

**Published:** 2014-05-09

**Authors:** XIAOSI HAN, WENBIN ZHANG, XIUHUA YANG, CRYSTAL G. WHEELER, CATHERINE P. LANGFORD, LU WU, NATALIA FILIPPOVA, GREGORY K. FRIEDMAN, QIANG DING, HASSAN M. FATHALLAH-SHAYKH, G. YANCEY GILLESPIE, L. BURT NABORS

**Affiliations:** 1 Division of Neuro-Oncology, Department of Neurology, University of Alabama at Birmingham, Birmingham, AL 35294-3410, USA; 2 Division of Neurosurgery, Department of Surgery, University of Alabama at Birmingham, Birmingham, AL 35294-3410, USA; 3 Division of Hematology/Oncology, Department of Pediatrics, University of Alabama at Birmingham, Birmingham, AL 35294-3410, USA; 4 Division of Pulmonary Medicine, Department of Medicine, University of Alabama at Birmingham, Birmingham, AL 35294-3410, USA

**Keywords:** Src family kinases, glioma stem cells, glioblastoma, dasatinib, migration, CD133

## Abstract

Src family kinases (SFKs) are highly expressed and active in clinical glioblastoma multiforme (GBM) specimens. SFKs inhibitors have been demonstrated to inhibit proliferation and migration of glioma cells. However, the role of SFKs in glioma stem cells (GSCs), which are important for treatment resistance and recurrence, has not been reported. Here, we examined the expression pattern of individual members of SFKs and their functional role in CD133^+^ GSCs in comparison to primary glioma cells. We found that Fyn, c-Src and Yes were robustly expressed in GSCs while Lck was absent. Knockdown of c-Src, Yes or treatment with the SFK inhibitor dasatinib inhibited the migration of GSCs, but had no impact on their growth or self-renewal. These results suggest that SFKs represent an effective target for GSC migration but not for their growth.

## Introduction

The Src family kinases (SFKs) are non-receptor tyrosine kinases that are membrane associated through a myristoylation site near their N-terminus. There are nine members in the family and five of which (Fyn, c-Src, Yes, Lyn and Lck) are expressed in human gliomas (1–5). Each member of the SFKs contains a unique N-terminal sequence, followed by four SH (Src homology) domains, and a C-terminal negative regulatory sequence. Structural study of c-Src has revealed that intra-molecular interactions occur between the phosphotyrosine 530 (pY530) in the C-terminus and the SH2 domain, and between the kinase domain and the SH3 domain, that cause the c-Src molecule to assume an inactive closed configuration (6). When pY530 is dephosphorylated, c-Src molecules become open and active, with the potential for autophosphorylation and phosphorylation of Src substrates. SFKs interact with multiple cell surface receptors including integrin, EGFR, PDGFR, VEGFR (2,3,7–9) and are activated rapidly upon receptor engagement resulting in the regulation of signaling events involving cell adhesion, migration, invasion, proliferation, apoptosis and angiogenesis (10,11).

The role of SFKs in glioma development and progression was demonstrated in transgenic mice of v-Src, a constitutively active mutant of Src (11–13). The v-Src transgenic mice, in which v-Src expression is under the control of the GFAP promoter, developed glial tumors with morphological and molecular characteristics that mimic human glioblastoma multiforme (GBM) (14,15). Although v-Src has not been reported in human glioblastoma, we now know that members of SFKs are effector molecules of EGFR, PDGFR, VEGFR and c-kit, many of which are overexpressed or constitutively activated in GBM (15). In addition, inhibition of SFKs by the tumor suppressor gene PTEN (phosphatase and tensin homologue deleted on chromosome 10) is abolished in gliomas due to mutation or loss of PTEN (16). Kinome profiling of clinical GBM specimens revealed that SFKs were highly activated (17,18). The SFKs specific inhibitor, PP2 or dasatinib, has been found to suppress migration, proliferation, and induce autophagy and cell death of glioma cells (15,17,19). These findings collectively suggest that SFKs represent an important target for glioma therapy.

Recent research has revealed that glioma stem cells (GSCs) are resistant to chemotherapy and radiation and are responsible for tumor recurrence (20,21). Effective therapies which target GSCs are needed. Consequently, we investigated the expression of SFKs in GSC and examined whether inhibitors of SFK could effectively inhibit the growth and migration of GSC. Since GSCs only account for a fraction of cells in a glioma tumor mass, high levels of SFKs in glioma tumors may not accurately reflect their levels in GSCs. In this study, we obtained GSCs and primary glioma cells (PGCs) from the same human GBM tumors xenografted in mice, and examined the expression and function of several members of SFKs in these two cell populations. We found that SFKs were highly expressed in GSCs and the expression patterns were different from that of PGCs. Fyn, Yes and c-Src were consistently expressed in both GSCs and PGCs while Lck was only expressed in PGCs. SFKs inhibitor dasatinib significantly inhibited migration of GSCs, but failed to inhibit their growth or self-renewal. These results suggest that SFKs represent an effective target for GSCs migration but not their growth.

## Materials and methods

### Culture of primary glioma cells and glioma stem cells from human GBMs xenografted in mice

All glioma xenografts were established by direct implantation of freshly resected human GBM tissue into the flanks of immunocompromised athymic nude (nu/nu) mice and maintained by serial transplantation as described previously (22). The University of Alabama at Birmingham Institutional Animal Care and Use Committee approved the use of all animal subjects. GSCs D456, JX6, JX10 and JX12 were cultured as we have described previously (22). To establish glioma primary and stem cell culture, xenograft tumors were harvested from the flank of mice and washed five times with PBS to remove excess blood. Tumors were separately minced finely with #11 scalpel blades and minced tumor was disaggregated in an enzyme solution [5 mg collagenase-I (Worthington Biochemical Corp., Lakewood, NJ, USA), 0.5% trypsin/0.53 mM EDTA (Gibco, Carlsbad, CA, USA), and 2.5 mg DNase-I (Worthington Biochemical Corp.)] in a sterile, vented, trypsinizing flask (20 min, room temperature). At 20 min intervals, approximately half of the cell suspension was removed and transferred to a centrifuge tube containing 0.5 ml of FBS. Fresh enzyme solution was added to the trypsinizing flask and the harvests were repeated four to five times, pooling the cells at each harvest. Cells were pelleted (200 × g, 8 min) and washed twice with DMEM/F12 (MediaTech) on ice. Dead cells were removed by density gradient centrifugation (1,500–1,800 × g, 20 min, 20°C) of 5×10^7^ cells in 35 ml carefully layered onto 10 ml of lymphocyte separation medium (Gibco). Cells collected at the interface were washed twice with DMEM/F12 to remove LSM. A portion of cells were added to glioma medium (DMEM/F12 50:50, 9% FBS, 1 mM glutamine, 10/ml penicillin and 10 *μ*g/ml streptomycin) for culture of primary glioma cells. The rest of cells were added to stem cell medium [NeuroBasal-A medium, supplemented with 1% B-27, 1X N-2 (Invitrogen, Carlsbad, CA, USA), bFGF and EGF (Peprotech, Inc., Rocky Hill, NJ, USA) at 10 ng/ml each, 10 U/ml penicillin and 10 *μ*g/ml streptomycine] and grow for 24–48 h before the CD133^+^ GSCs were isolated by FACS (see below). The isolated CD133^+^ GSCs were grown in stem cell medium exchanged every 3–4 days, and passaged with Accutase (Invitrogen) weekly.

### FACS analyses and separation of CD133^+^ glioma stem cells

CD133^+^ GSCs in glioma spheres were analysed and isolated by FACS. Briefly, spheroids were harvested from culture, pelleted by centrifugation (300 × g, 8 min), resuspended in 1 ml of Accutase and incubated (37°C, 5–10 min) with occasional shaking. The cell suspension was gently triturated several times with a wide bore pipette to dissociate any remaining glioma spheres. Cells were washed twice with DMEM/F12 50:50 and counted. Viable cells (4×10^6^) were resuspended in 80 *μ*l of FACS buffer containing ice-cold PBS with 5% FBS and 0.1% of sodium azide. FCR blocking reagent (20 *μ*l, Miltenyi Biotech) and allophycocyanin (APC)-conjugated anti-CD133/2 antibody (10 *μ*l, Miltenyi Biotech) were added. Cells were incubated (15 min, 4°C), washed and resuspended in 1 ml ice-cold PBS with 0.1% sodium azide. Cells were analyzed immediately by FACS and CD133^+^ cells isolated at UAB Flow Cytometry Core Facility. The side-scatter versus forward light scatter profiles of a control sample was used to set gates. Data of FACS were analyzed using Flo-Jo software and results were expressed as a percentage of gated cells for each cell type identified by antibody binding.

### GSC self-renewal and differentiation assay

For GSC self-renewal assay, a single cell suspension was obtained from a single sphere by repetitive pipeting up and down. Cells were diluted in stem cell medium at a concentration of 10 cells/ml, and 100 *μ*l was plated into each well of a 96-well plate and grown for 1 week when 100 *μ*l of fresh stem cell medium was added. Glioma spheres in each well were counted 2 weeks after plating. For differentiation assay, GSCs were dispersed into single cell suspension and grown on cover slips in DMEM/F12 (50:50) supplemented with 2% FBS. Cells were grown for 7 days before staining with antibodies against glial marker GFAP (Rabbit polyclonal IgG; Dako, Carpinteria, CA, USA), neuronal marker βIII-tubulin (mouse monoclonal IgG; Chemicon, Temecula, CA, USA) and oligodendrocytic marker MBP (myelin basic protein, mouse monoclonal IgG; Abcam, Cambridge, MA, USA). Glioma spheres were immunostained with anti-Sox2 antibody (rabbit polyclonal IgG; Santa Cruz Biotechnology, Inc., Santa Cruz, CA, USA). Alexa-488 goat anti-rabbit IgG and Alexa 595 goat anti-mouse IgG (Invitrogen) were used as secondary antibody for immuno labeling. Cells were visualized under fluorescent microscopy.

### Immunoblotting

Cultured cells were lysed in M-PER mammalian protein extraction buffer (Thermo Scientific) in the presence of 1X Halt proteinase and phosphatase inhibitor cocktail (Thermo Scientific) (100 *μ*g/ml aprotinin, 10 *μ*g/ml leupeptin, 2 mg/ml AEBSF hydrochloride, 50 *μ*g/ml Bestatin, 200 *μ*g/ml E-64, 100 mg/ml EDTA, 10 *μ*g/ml Pepstatin A). Protein concentrations were determined by BCA protein assay kit (Pierce Biotechnology, Inc., Rockford, IL, USA). Equivalent amounts of protein (20 *μ*g) were resolved on an 8–16% gradient Precise Protein Gel (disulfide-reduced SDS-PAGE gel; Thermo Scientific), transferred to a nitrocellulose membrane (Millipore, Billerica, MA, USA), blocked with 5% non-fat milk in TBST (1 h, 22°C), and reacted with the primary antibody at 4°C overnight followed by a secondary antibody conjugated to horseradish peroxidase (Sigma, St. Louis, MO, USA). Membrane-bound antibodies were detected by enhanced chemiluminescence (BD Biosciences, Franklin Lakes, NJ, USA). Relative band intensities were determined by averaging the intensity readings from three independent experiments using QuantityOne software with a VersaDoc imaging system (Bio-Rad, Hercules, CA, USA). Src family member-specific rabbit anti-Lyn, anti-Fyn, anti-Yes and anti-Lck IgG were purchased from Santa Cruz Biotechnology, Inc., and rabbit anti-c-Src IgG, mouse anti-Src monoclonal antibody (clone GD11) and mouse anti-Src family (WTAPE) antibody were purchased from Millipore. Rabbit anti-phosphorylated (pY416)-Src polyclonal antibody was from Cell Signaling Technology, Inc. (Danvers, MA, USA) and mouse anti-actin was from Sigma. Each antibody was used from one lot and the optimal concentration was determined. The following antibody concentrations were used for blotting: rabbit anti-c-Src, anti-Lyn and anti-Fyn IgG (0.1 *μ*g/ml), rabbit anti-Yes and anti-Lck IgG (0.2 *μ*g/ml), rabbit anti-phosphospecific Src IgG (pY416-Src, 0.01 *μ*g/ml) and mouse anti-actin (0.1 *μ*g/ml).

### Proliferation and cell viability assay

PGCs in glioma medium with 2% FBS were seeded at a density of 2.5×10^3^ cell/well in a 96-well plate overnight and treated with dasatinib (100 *μ*M) the next morning. Medium was changed every 2 days and viable cells were determined at day 2, 4 and 6 after treatment. For GSCs, single cells obtained from neurospheres were cultured in T-25 flasks in the presence and absence of dasatinib, samples of 100 *μ*l were drawn with a large bore pipette tip and subjected to cell viability assay on day 2, 4 and 6 after treatment. In this assay, cellular ATP levels were assessed using the ViaLight Plus kit (Lonza, Rockland, ME, USA). This assay measured ATP levels in the lysates by a luciferase-catalyzed production of light from luciferin. Emitted light, which is linearly related to the amount of ATP present in the lysates, was measured in a Spectrafluor Plus machine (Tecan, Männedorf, Switzerland). The relative cell amount represents the averages of three experiments in triplicates. In addition, viable cells of GSCs and PGCs under each growth condition were counted using hemocytometer by trypan blue exclusion. PGCs (100,000) were plated into a T-25 flask in glioma medium containing 2% FBS with or without dasatinib, and cells were counted with trypan blue exclusion on day 2, 4 and 6. For GSCs, glioma spheres were collected, digested with Accutase into single cells, and counted on day 2, 4 and 6 of the dasatinib treatment.

### Migration assay

Two-well Boyden-type chambers with 8-*μ*m pore non-coated polycarbonate filters (Costar) were coated on the lower filter surface with laminin (10 *μ*g/ml, Invitrogen) at 37°C overnight, followed by blocking with 2% BSA in PBS (37°C, 1 h). Migration assay buffer (DMEM/F12 50:50 with 0.1% BSA, 1 mM L-glutamine) supplemented with EGF and FGF (20 ng/ml) as a chemoattractant was added to the bottom chamber. PGCs (harvested with Versene, Invitrogen) and GSCs (obtained by digesting the glioma spheres with acccutase) were washed and resuspended in migration assay buffer at 200,000 cells/ml. Cell suspension aliquots (100 *μ*l) were loaded onto the upper chamber and incubated at 37°C (5% CO_2_). At 3 h, cells on the upper filter surface were removed by wiping with Q-tips and cells on the lower filter surface were fixed (4% buffered paraformaldehyde, 10 min), washed, stained (1% crystal violet, 15 min) and quantified by counting cells in 5 random high power fields (1-mm^2^ fields in a 1-cm grid). Cell numbers were averaged from three independent experiments. Some of the filters with migrated cells fixed on the lower surface were used for immunofluorescent staining (see below).

### Immunofluorescent staining

The cells were allowed to migrate onto the laminin coated filter surface as above. The filters were fixed by paraformaldehyde, blocked in PBS containing 5% each of BSA and horse serum (25°C, 60 min) and reacted with rabbit anti-CD133 polyclonal IgG (Abcam) at 4°C for overnight. Cells were washed, incubated with AlexFluor-488 conjugated goat anti-rabbit IgG (1:1000, 22°C, 60 min). After rinsing in PBS, cells were stained with DAPI (50 ng/ml in PBS, 2 min) and visualized under an Olympus fluorescence microscope equipped with a digital camera. Monochrome images from each color channel were acquired separately and then merged. Fluorescence images were processed using Adobe Photoshop (Adobe Systems, San Jose, CA, USA).

### siRNAs and transfection

Control siRNA or siRNAs (100 pmol) specific for human c-Src, Yes and Fyn (SMARTpool; Dharmacon, Lafayette, CO, USA) was transfected into 4×10^5^ GSCs by using Lipofectamine 2000 (Invitrogen) according to protocol recommended by the manufacture. Cells were plated and cultured for 72 h prior to immunoblot analysis for target knock down and migration assay.

### Statistical analysis

Two-tailed Student’s t-tests were used to determine the statistical significance between two groups (Sigma Plot 2000; SPSS Inc., Chicago, IL, USA), and were expressed as mean ± standard errors (SE). All experiments were repeated at least three times. A P-value of <0.05 was considered to be statistically significant.

## Results

### Culture of glioma stem and primary glioma cells

**Figure 1.**
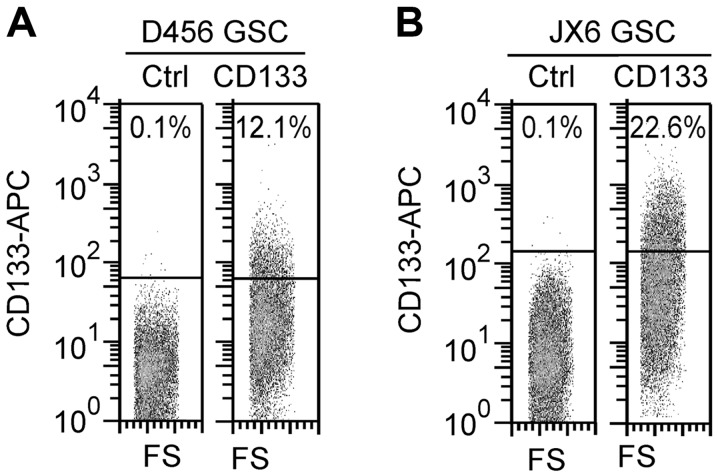
CD133^+^ population in GSC D456 and JX6. Single cells suspensions were obtained from glioma spheres and labeled with control antibody (mouse IgG-APC) or anti-CD133-APC. CD133^+^ GSCs were analyzed by FACS, and false positive in the controls was set to 0.1%. (A) CD133^+^ GSCs represent 12.1±1.04% in D456. (B) CD133^+^ GSCs account for 22.6±1.51% in JX6.

Four pairs of GSCs and PGCs were obtained from four human GBM xenografted in mice (D456, JX6, JX10 and JX12) as described previously (22). The CD133^+^ population in GSC D456 and JX6 reached a steady level of 12.1±1.04% and 22.6±1.51%, respectively, after 4 passages and remained stable for at least up to 8 passages (Fig. 1). The CD133^+^ cells in GSC JX12 and JX10 were determined to be 36.2 and 9.1%, respectively, at passage 4, but the two cell lines decreased their CD133^+^ populations with each passage and became growth arrested after 8 passages. The GSC JX10 and JX12 were excluded from further analysis due to instability of CD133^+^ population. The results of CD133^+^ populations in these GBM xenografts are consistent with the previous data (22). For culture of PGCs, cells obtained from the GBM xenografts were grown in regular medium containing 9% FBS. PGCs grew as a monolayer and the CD133^+^ populations were <2% (see data below).

**Figure 2.**
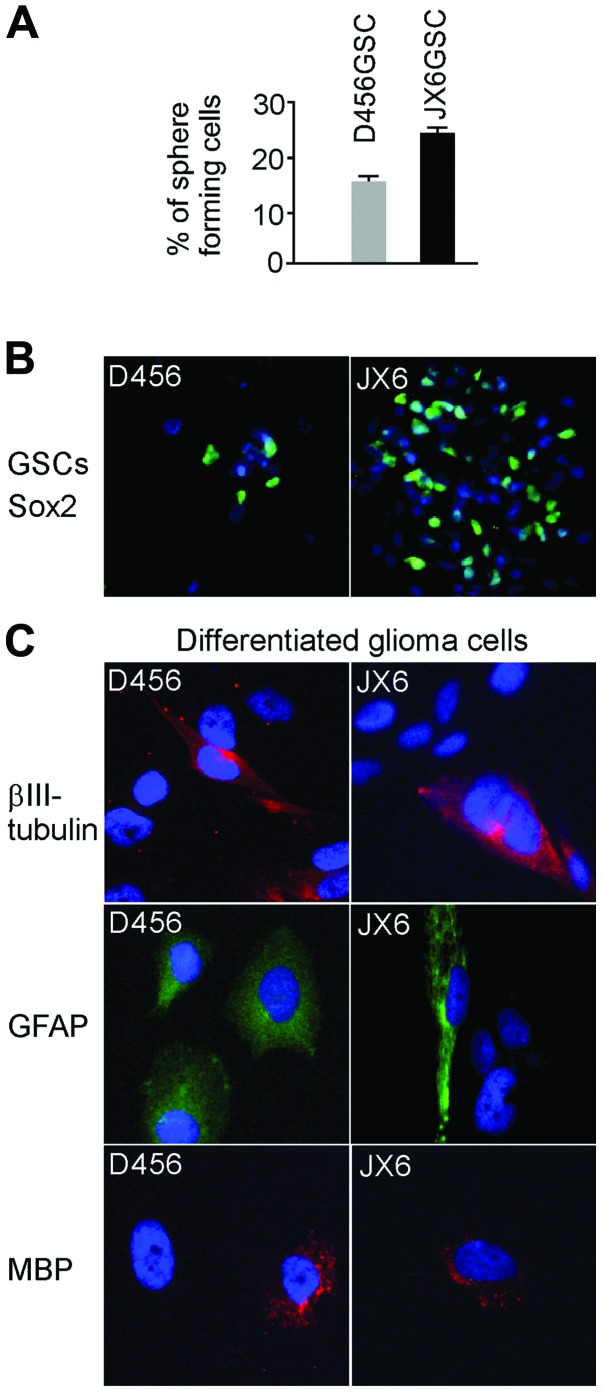
GSC D456 and JX6 could self-renewal and differentiate into cells with neuronal, astrocytic and oligodendraglial markers. Single cells obtained from a single GSC sphere were grown for 2 weeks before spheres were counted. (A) Sphere forming rates were 16±0.9% and 24±1.2% for GSC D456 and JX6, respectively. (B) Glioma spheres were stained for GSC marker with anti-Sox2 and the result showed some cells in the glioma spheres are positive. (C) After differentiation in medium containing 2% FBS for 10 days, cells positive for marker of astrocytes (GFAP), oligodendrocytes (MBP) and neurons (βIII-tubulin) were shown.

**Figure 3.**
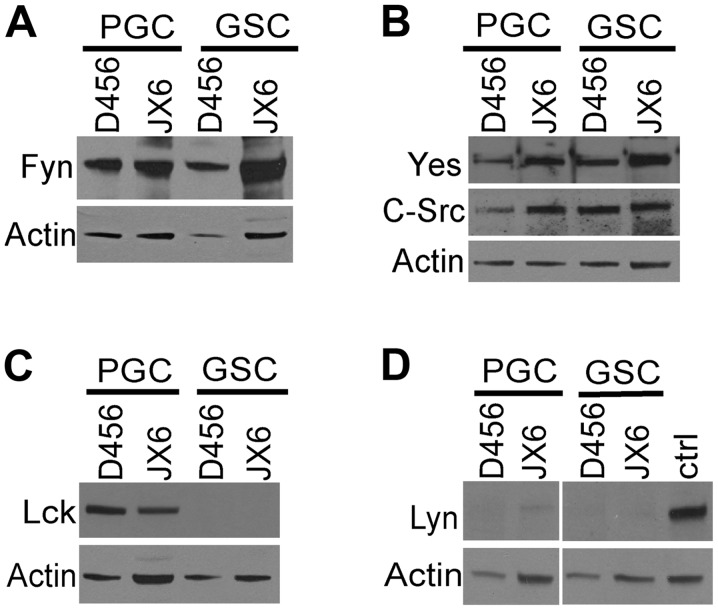
Expressions of Fyn, Yes, c-Src, Lyn and Lck in PGCs and GSCs. Equal amounts of protein (20 *μ*g) were loaded into each lane and were blotted with anti-Fyn, Yes, c-Src, Lck, Lyn and actin for loading control. Fyn, Yes and c-Src were expressed in all the GSCs and PGCs (A and B). (C) LCK was expressed in PGCs but absent in GSCs. (D) Lyn was low in PGCs and GSCs D456 and JX6 (extract from PGCs JX10 was used as a positive control).

The self-renewal capacity of the GSCs was determined by the self-renewal assay. We found that 16±0.84% of single cells from GSC D456 and 24±1.2% of cells from GSC JX6 formed spheres (Fig. 2A). The cells in daughter spheres of both GSC D456 and JX6 had the same potential to form spheres as their parent. Next, we determined the differentiation capacity of these GSCs. Cells in glioma spheres were immunostained with anti-Sox2 (a glioma stem cell marker) and the results are shown (Fig. 2B). Cells from glioma spheres were grown in serum-containing medium to induce differentiation before they were stained for specific markers for astrocytes (GFAP), oligodendrocytes (MBP) and neurons (βIII-tubulin) (Fig. 3C). All three lineages of neuroepithelial cell including astrocytes, oligodendrocytes and neurons were present. The glioma spheres were tumorigenic as these GBM xenografts were maintained by sequential re-inoculation of the cells from glioma spheres obtained from the immediate prior generation of xenografts. These results demonstrated that cells grown in stem cell medium that had stable CD133 expression contained the ability to self-renew and differentiate, both features of GSCs.

### SFKs are expressed and active in GSCs

The expression of SFKs in GSCs was determined in comparison to that of the paired PGCs which served as references. We harvested the glioma spheres and the PGCs and determined Fyn, Yes, c-Src, Lyn and Lck (Fig. 3). Fyn, Yes and c-Src were detected in both GSCs and PGCs (Fog. 3A and B). Expression of Lck showed a differential pattern, it was absent in both GSCs whereas it was present in the PGCs (Fig. 3C). Expression of Lyn was very low in the GSCs and PGCs (Fig. 3D). These results demonstrate that Fyn, Yes and c-Src were the three major SFKs present in D456 and JX6 GSCs.

**Figure 4.**
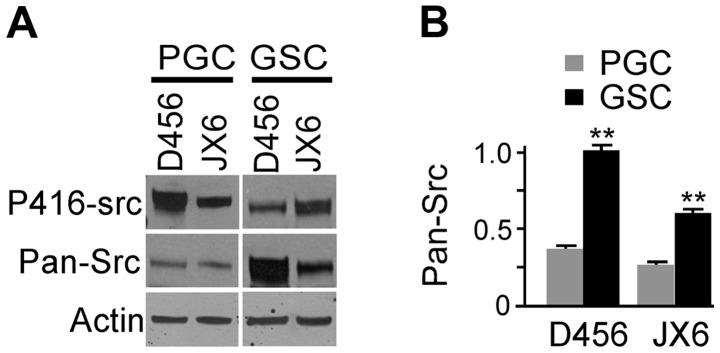
SFKs are expressed at high levels in GSCs. The levels of SFKs and active SFKs were assessed using anti-pan-Src and anti-p416-Src (active Src). (A) Active form and total SFKs (pY416-Src) in PGCs and GSCs were shown; the blot was probed with anti-actin for loading control. (B) The levels of SFKs in GSCs were significantly higher than that of PGCs as determined by chemiluminescence intensity and normalized against actin. The results represent the averages of three independent experiments; ^**^P<0.01.

The active SFKs in GSC were evaluated by detecting the amount of pY416 residue with anti-pY416-Src (Fig. 4A, upper panel). Anti-pY416-Src reacts with multiple members of SFKs (Fyn, Yes, c-Src, Lyn and Lck) when activated and phosphorylated at equivalent sites. p-416-Src was detected in all cells and it appeared higher in PGCs than that of corresponding GSCs. Total SFKs were assessed in GSCs by immunoblotting using mouse anti-Src family (WTAPE) antibody, which react with multiple members of SFKs including Src, Yes, Fyn and others (Fig. 4A, middle panel). Anti-Src family antibody detected bands around ∼60 kDa which represented members of SFKs. The expression levels of SFKs (pan-src) were normalized by β-actin and the relative levels of SFKs in each GSCs and PGCs are shown (Fig. 4B). The levels of pan-Src in GSCs were significantly higher than that of their paired PGCs. Taken together, the results indicate that the total SFKs are expressed at higher levels in GSCs compare with PGCs.

### Dasatinib effectively penetrates into glioma spheres and inhibits SFKs in GSCs

**Figure 5.**
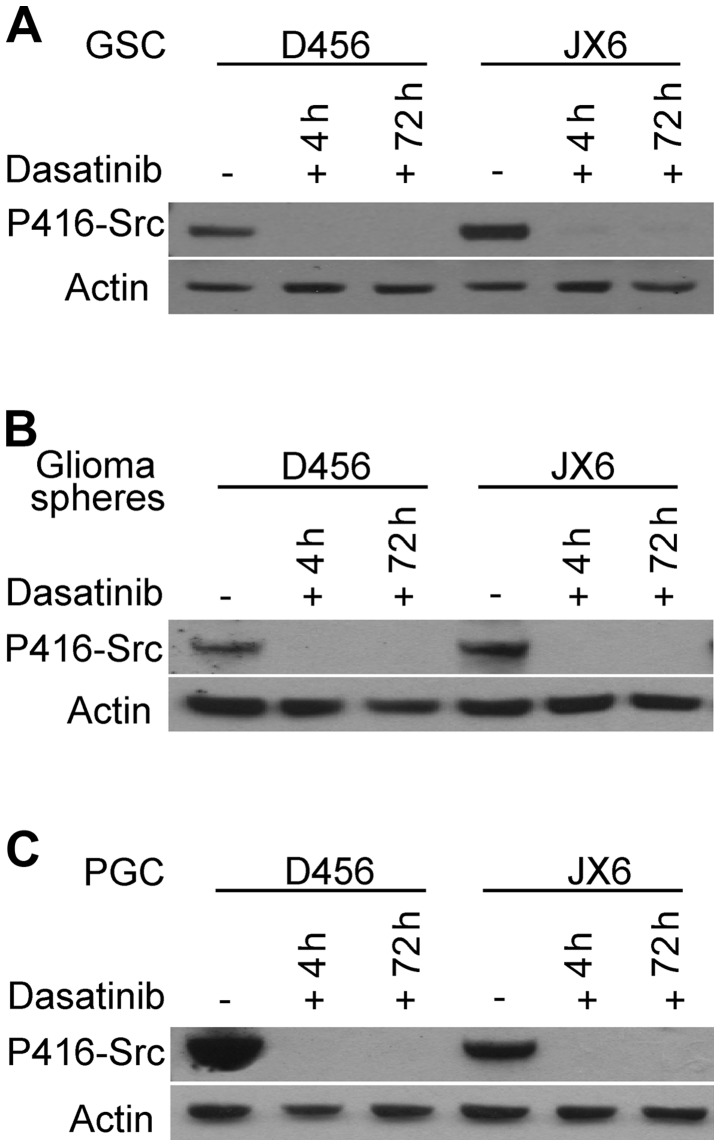
Dasatinib inhibited SFKs in GSCs, large glioma spheres and PGCs. (A) Single GSCs prepared from neurospheres were grown in a 25-cm^2^ flask in the presence of dasatinib (100 nM). Cellular protein lysates were prepared at 4 and 72 h after treatment and reacted with anti-p416Y-Src. (B) Single GSCs suspensions were grown for 14 days without splitting to form large neurospheres. Neurospheres were treated with dasatinib for 4 and 72 h, respectively, and cellular proteins were probed with anti-p416Y-Src. (C) p416-Src was completely inhibited by dasatinib treatment in PGCs.

It is well established that dasatinib inhibits SFKs in many cell lines that grow as monolayer or single cell suspension, including glioma cells (8,15,17,19). However, it is unclear whether dasatinib can effectively inhibit the SFKs in GSCs because these cells grow as neurospheres that may not be well penetrable by dasatinib. In addition, GSCs may employ mechanisms such as the ABC transporter to eliminate intracellular small molecules as seen in normal neural stem cells (23). To address the impact of dasatinib on the growth of GSCs, we first examined whether dasatinib could inhibit the activation of SFKs in GSCs. GSCs were grown in stem cell medium without or with dasatinib at 100 nM, a concentration similar to the clinical plasma trough concentration at therapeutic dose (24). At 4 and 72 h of the treatments, the cell lysates were prepared for analyzing pY416-Src, the activated form of SFKs. As shown in Fig. 5A, pY416-Src was undetectable 4 h after addition of dasatinib, and the inhibitory effect persisted 72 h afterward in the GSCs. The results demonstrate that dasatinib effectively inhibited SFKs in GSCs grown as single cells or small neurospheres. We then examined whether dasatinib would be able to penetrate into large neurospheres. To do this, single stem cells suspension were allowed to grow for 14 days without splitting to form large glioma neurospheres before dasatinib treatment. As shown in Fig. 5B, dasatinib effectively abolished the pY416-Src in all the glioma neurospheres. As a control, we examined the effect of dasatinib on p416-Src in PGCs and found that it was completely inhibited as expected (Fig. 5C). These results demonstrated that dasatinib, at a clinical therapeutic serum concentration, is able to effectively penetrate into GSCs and inhibit SFKs.

### Inhibition of SFKs by dasatinib fails to suppress the growth or self-renewal of GSCs

**Figure 6.**
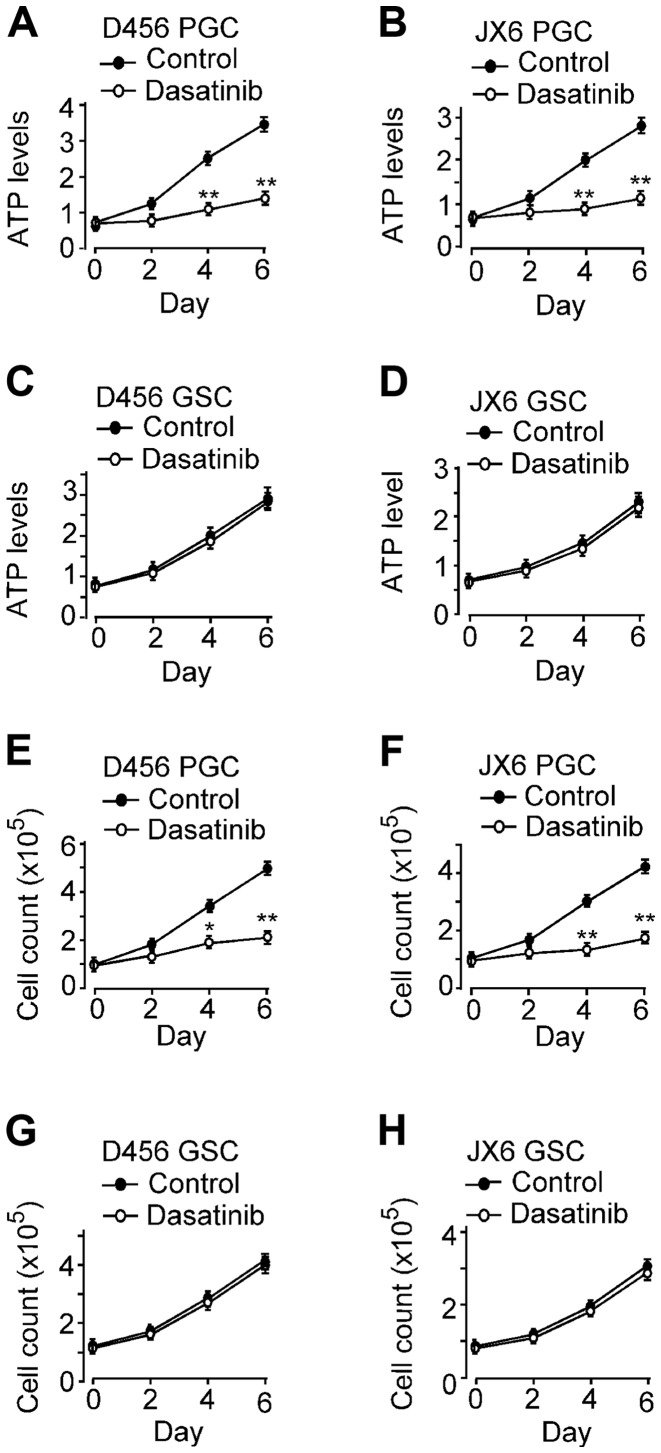
SFKs inhibition by dasatinib inhibited the growth PGCs but not GSCs. Equal numbers of cells were plated into each well of a 96-well plate with or without dasatinib (100 nM) and viable cells were determined on day 2, 4 and 6. (A and C) Dasatinib treatment significantly inhibited the growth of PGS D456 and JX6. (B and D) Dasatinib treatment had no effect on the growth of GSC D456 and JX6. (E–H) The effects of dasatinib on the growth of PGCs and GSCs were independently determined by cell counting with trypan blue exclusion under the same growth condition. The assays were performed in triplicates and repeated 3 times; ^*^P<0.05, ^**^P<0.01.

It has been known that dasatinib inhibits the growth of PGCs, glioma cell lines and gliomas in animal models (17,18,25), however, the effects of dasatinib on the growth and self-renewal of GSCs have not been reported. The effect of SFKs inhibition by dasatinib on the growth of GSCs was assessed in GSC D456 and JX6 in comparison to the paired PGCs. We plated 1500 cells into each well of a 96-well plate in culture medium containing 2% FBS with or without dasatinib (100 nM). Viable cells were determined on day 2, 4 and 6 of the treatment (Fig. 6A and B). As expected, the growth of PGC D456 and JX6 were significantly inhibited by dasatinib. By day 6, PGCs D456 and JX6 grew by 380±18% and 322±16%, respectively, in control, whereas cells in the dasatinib containing medium grew by 105±4.8% and 74.1±3.8%, respectively. These results are consistent with the previous reports that dasatinib inhibits the growth of glioma cells (15,17,19). The effect of dasatinib on the growth of GSC D456 and JX6 were determined by cell viability assay (Fig. 6C and D). The growth of GSC D456 and JX6 cell lines was not affected by dasatinib. By day 6, GSC D456 and JX6 grew by approximately 260±11% and 210±9%, respectively in the controls. The growth rate was not significantly affected by treatment of dasatinib. The effect of dasatinib on cell proliferation was further confirmed by cell counting. GSCs and PGCs grown under the same conditions as above were counted with trypan blue exclusion on day 2, 4 and 6 (Fig. 6E–H). By day 6, the PGC D456 and JX6 in control medium grew by about 370±16% and 320±13%, respectively, but the cells in dasatinib containing medium grew only by about 120±4.9% and 70±2.8%, respectively. However, the growth of GSC D456 and JX6 GSC was unaffected by treatment of dasatinib. These results were consistent with those obtained by cell viability assay. Taken together, the data demonstrated that the growth of GSCs was not affected by SFKs inhibition as opposed to that of PGCs, suggesting that SFKs are not critical in the growth of GSCs.

### Inhibition of SFKs by dasatinib did not change the proportion of CD133^+^ GSCs in glioma spheres

To assess whether the proportion of CD133^+^ GSCs in glioma spheres were affected by exposure to dasatinib, we examined the population of CD133^+^ GSCs by FACS. The CD133^+^ cell population in GSC D456 and JX6 were 12.1 and 22.6%, respectively (Table I). The proportions were essentially the same after treatment with dasatinib for 14 days in both GSCs. The results support that the growth of GSCs, in contrary to PGCs, are intrinsically insensitive to SFKs inhibition by dasatinib. For comparison, we treated PGCs with 100 nM dasatinib for 14 days and determined the CD133^+^ cell population prior and after treatment (Table I). We found that the CD133^+^ population expanded in D456 and JX6 PGCs, likely as a result of preferential inhibition of differentiated PGCs.

### Inhibition of SFKs by dasatinib significantly suppresses the migration of GSCs

**Figure 7.**
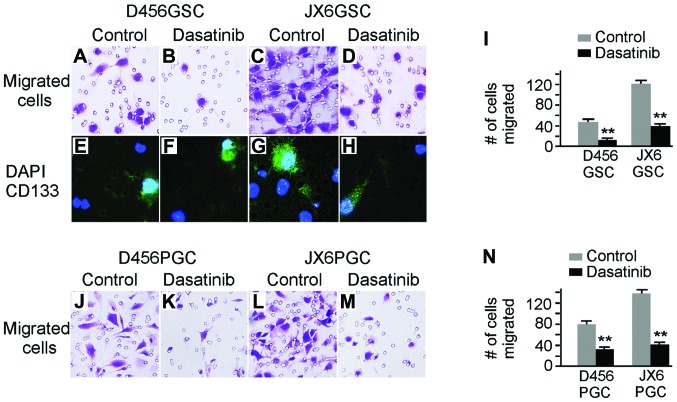
Dasatinib inhibited migration of GSCs. GSCs were loaded into laminin coated transwells in the presence or absence of dasatinib, and were allowed to migrate for 3 h toward chemoattractants (EGF and FGF for GSCs, serum for PGCs). (A–D) The migrated cells were fixed with 4% paraformalaldehyde and stained with crystal blue. (E–H) Migrated cells on the transwell membrane were stained with rabbit anti-CD133 antibody followed by Alexor-488 conjugated goat anti-rabbit IgG. The cell nuclei were counter stained with propidium iodide. (I) The migrated GSCs were counted in 10 high power fields and the average numbers of cells per high power field in the presence or absence of dasatinib were shown. The numbers represent averages of three independent experiments in triplicates. (J–M) The effects of dasatinib on migrations of PGCs are shown. (N) The migrated PGCs in the presence or absence of dasatinib were quantified. The numbers represent averages of three independent experiments in triplicates; ^**^P<0.01.

One of the most important functions of SFKs is its role in cancer cell migration and invasion. It is technically difficult to examine the migration of free floating GSCs. Recent research demonstrates that GSCs grow as a monolayer on laminin or other extracellular matrix coated flask without changing the stem cell properties (26,27). When the two GSC cell lines (D456 and JX6) were plated in flasks pre-coated with laminin (10 mg/ml, 37°C overnight), the GSCs grew as monolayer and were readily reversible to spheres in the non-coated flask. To evaluate whether SFKs affected GSC migration, cell migration assay was performed on laminin coated transwells and the cells that migrated in the control and dasatinib treated samples were visualized and counted (Fig. 7). The migration of D456 and JX6 GSCs were significantly inhibited by dasatinib treatment (Fig. 7A–D). To confirm that the migrated cells contain CD133^+^ GSCs, we immunostained the migrated cells with rabbit anti-CD133 antibody followed by Alexor-488-conjugated goat anti-rabbit IgG (Fig. 7E–H). The CD133^+^ GSCs were present in the migrated cell population regardless of dasatinib. The inhibitory effects of dasatinib on migration were quantified and shown in Fig. 7I. As a control, we examined the effect of dasatinib on migration of PGCs, and observed reduced migration (Fig. 7I and N).

### c-Src and Yes are involved in the migration of GSCs

**Figure 8.**
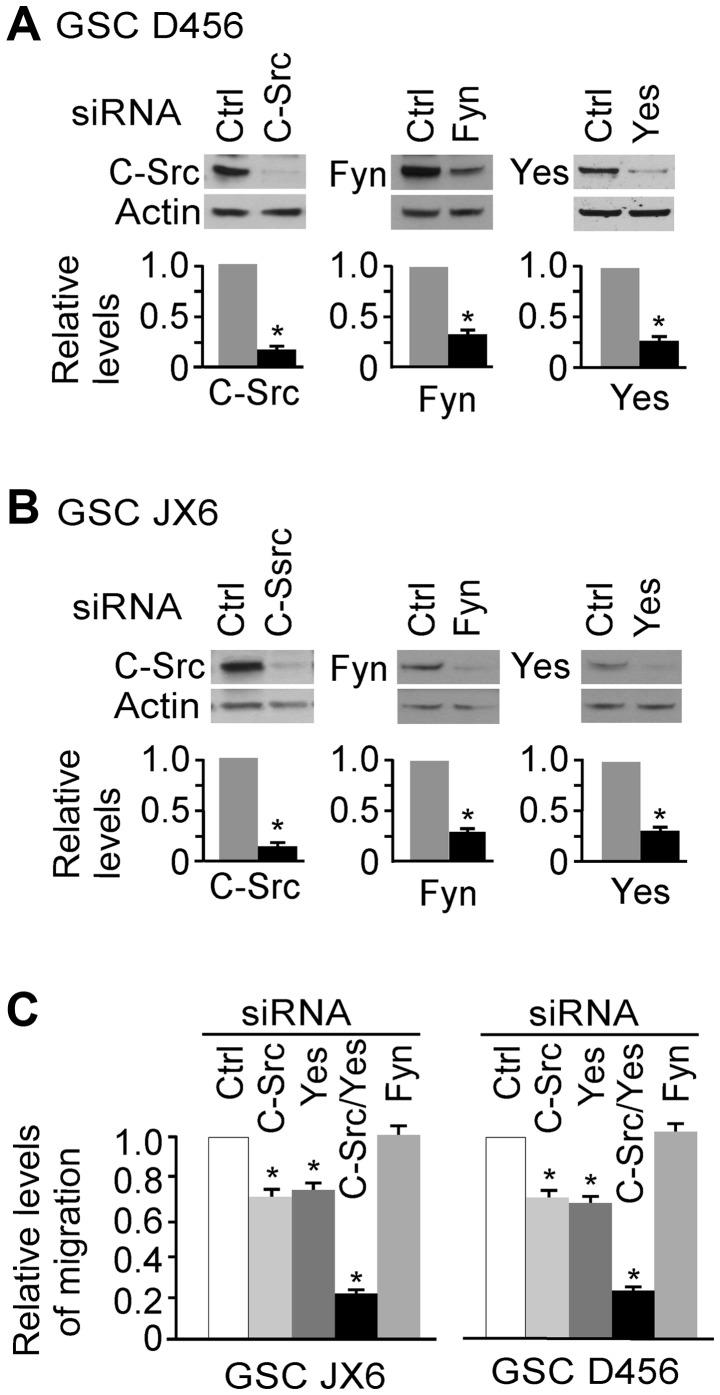
Knockdown of c-Src and Yes by siRNA significantly inhibited migration of GSCs. Control and siRNAs (100 pmol) for human c-Src, Yes and Fyn was transfected into 4×10^5^ GSCs using Lipofectamine 2000. Cells were grown in 6-well plates for 72 h prior to immunoblot analysis for target knock down. (A and B) c-Src, Yes and Fyn were knocked down significantly in each of the cell lines. (C) Cell migrations on a laminin coated transwell are shown. The knockdown of either c-Src or Yes significantly decreased the migration of GSCs, but the knockdown of Fyn had no effect. Combined knockdown of both c-Src and Yes inhibited GSCs migration additively. The numbers represent averages of three experiments in triplicates; ^*^P<0.01.

To dissect the role of c-Src, Yes and Fyn in the migration of GSCs, we knocked down each of these three targets by siRNA and the results are shown in Fig. 8A and B. c-Src, Fyn and Yes were knocked down by 86, 72 and 75%, respectively in D456 GSCs, and by 82, 71 and 70%, respectively in JX6 GSCs. Cells with target knockdown were used for transwell migration assays and the results are shown in Fig. 8C. Knocking down of either c-Src or Yes significantly inhibited the migration of GSCs in each of the GSCs. However, knockdown of Fyn did not alter the migration capacity of these cells. Furthermore, simultaneous knockdown of Yes and c-Src inhibited cell migration by approximately 80% in both GSCs. These results indicate that c-Src and Yes are directly involving in migration of these cells on a laminin coated surface.

## Discussion

SFKs are important kinases involved in glioma cell migration and invasion, and SFKs inhibitors have been in investigation for anti-glioma therapy. Recent data indicate that GSCs are important for glioma initiation, treatment resistance and recurrence. In this study, we demonstrate that several members of SFKs (Fyn, c-Src and Yes) are expressed and active in GSCs. Inhibition of SFKs by dasatinib has no effects on the growth or self-renewal of GSCs, but significantly inhibited the migration of GSCs. Knockdown of c-Src and Yes significantly inhibited migration of GSCs indicating that these SFKs are important for GSCs migration.

Five members of SFKs, Fyn, c-Src, Yes, Lyn and Lck, were reported to be expressed in glioma cells (1–5), and our results in PGCs are consistent with these reports. Previous studies demonstrated that c-Src and Fyn interact with and phosphorylate the cytoplasmic tail of CD133 in medulloblastoma stem cells (28), and it is possible that Fyn, c-Src and Yes directly interact with CD133 in GSCs; however inhibiting SFKs by dasatinib did not alter CD133 expression patterns in GSC lines. Interestingly, Lck appears to be expressed in PGCs but not in the paired GSCs. A recent study showed that Lck is activated in the glioma cell lines U87 and U373 after exposure to radiation and the activation is important for expansion of GSCs after radiation treatment (29). Lck may play a specific role in radiation induced expansion of GSCs, however, our results suggest that Lck activity is not necessary for the maintenance of GSCs.

Dasatinib is an ATP-competitive, dual SFKs/ABL inhibitor, and it inhibits all members of SFKs including c-Src, Lck, Fyn and Yes (IC_50_<1.1 nM) (30–32). At higher concentrations (3–28 nM), dasatinib also inhibits Abl, PDGFR, c-kit and EphA2 (31). Previous studies demonstrated that PDGFRs are frequently expressed in malignant glioma (33,34) as well as EphA2 (35,36). However, expression of c-kit and c-abl is rarely identified in glioblastoma (34). The concentration we used is based on clinical serum trough level (24) and is a concentration that will inhibit all the above targets if present. It should be noted that the concentration of dasatinib in cerebrospinal fluid (CSF) at a clinically therapeutic dose is undefined. However, dasatinib likely reaches a therapeutic concentration in CSF because it has demonstrated remarkable activity in patients with CNS metastases from Ph^+^ leukemia (37).

Inhibition of SFKs by dasatinib significantly suppressed proliferation of PGCs but had no significant inhibitory effect on the growth of GSCs. The mechanism for GSC insensitivity to dasatinib-mediated inhibition of SFKs and possible other receptor tyrosine kinases is unclear. Previous studies demonstrated that GSCs are intrinsically more resistant to chemotherapeutic agent, temozolomide, as well as radiation treatment (20,21). Insensitivity to SFK inhibition is likely yet another intrinsic property of at least some GSCs. GSCs are maintained and regulated in niches close to blood microvessels (20,21), and various factors such as cell-to-cell communication, various secreted factors and signals, and oxygen tension all influence whether GSCs self-renew, proliferate or migrate (38). These niches are rich in laminin which strongly interacts with α6, an integrin receptor highly expressed in normal neural stem cells as well as GSCs (39,40). The laminin/α6 integrin mediated signaling could be inhibited by dasatinib leading to inhibition of migration of GSCs. The inhibition of anchorage/migration has no direct effect on the ability of GSC to self-renew or proliferate. Dasatinib significantly inhibited the growth of PGCs and the growth inhibition was time sensitive. Freshly isolated primary cell lines from xenografts and the first few passages appeared more sensitive to dasatinib treatment than did later passages (data not shown). While the mechanism is unknown, it is clear that cells isolated from tumors change dramatically after a few passages in serum containing medium when compared to cells grown in defined serum-free medium designed to support GSCs (41). It is likely that the cells are initially under stress after disaggregation and an additional stress superimposed by treatment of dasatinib resulted in a greater effect on cell growth and survival. Dasatinib significantly inhibited the migration of both GSC lines in transwell migration assays. The effect is mediated at least in part by c-Src and Yes because the knockdown either molecule decreased the migration capacity of GSCs. It would be useful to know whether α6β1 preferentially interacts with c-Src or Yes in laminin mediated anchorage and migration of GSCs.

In conclusion, our results demonstrate that SFKs are expressed and active in GSCs which are important for migration of GSCs. Inhibition of SFKs by dasatinib inhibited the growth of PGCs but was ineffective at inhibiting the growth of GSCs. The results suggest that SFK inhibitors such as dasatinib remain valuable agents for targeting some glioma cells; however, SFK inhibitor alone is likely to have a limited effect on growth and self-renewal of GCCs.

## Figures and Tables

**Table I.** Dasatinib treatment did not change the proportion of CD133^+^ populations in GSCs.

Dasatinib	CD133^+^ GSCs (%)	
	−	+
GSC D456	12.1±1.04	12.9±1.14
GSC JX6	22.6±1.51	23.2±1.90
PGC D456	0.72±0.09	3.76±0.24^a^
PGC JX6	0.52±0.08	1.62±0.12^a^

a P<0.01.
